# A Rare Complication of Internal Jugular Vein Cannulation Following Subcutaneous Implanted Port Placement

**DOI:** 10.7759/cureus.24862

**Published:** 2022-05-09

**Authors:** Anjo Chacko, Amber Kean, Farrah Benoit, Vijaykumar Patel

**Affiliations:** 1 General Surgery, Wellstar Atlanta Medical Center, Atlanta, USA; 2 General Surgery, Wellstar Atlanta Medical center, East Point, USA

**Keywords:** ultrasound-guided, port-a-cath, video-assisted thoracoscopic surgery (vats), hemothorax, internal jugular vein catheterization

## Abstract

Port-a-Cath also known as a subcutaneous implantable catheter is a common device used in patients undergoing drug infusions. Port-a-Cath placements are widely used among cancer patients who need multiple intravenous infusions with chemotherapeutic agents. The surgical approach to implanting a Port-a-Cath is associated with risks and benefits; however, it may also be associated with serious complications. We describe a rare case of a large right-sided hemothorax following right internal jugular vein cannulation after Port-a-Cath placement. We discuss possible causes of hemothorax in this patient and describe possible factors such as abnormal anatomy of vessels and body habitus contributing to this complication. We also highlight the use of imaging such as ultrasound-guided techniques and the importance of postoperative chest radiographs to screen for possible complications.

## Introduction

Subcutaneously implanted port placements with a Port-a-Cath device have become a common procedure in the management of patients with cancer. Since the introduction of this device, there has been improved efficiency and simplification in the administration of chemotherapeutic agents as well as venous blood sampling in patients [1]. Complications following port placement surgery can be of various etiologies and can be divided into early and delayed complications based on the time of onset. Early complications include venous malpositioning, pneumothorax, hemothorax, thoracic duct injury, or even cardiac tamponade [2-4]. Delayed complications include infection, catheter thrombosis, vessel thrombosis and stenosis, catheter fracture with extravasation, or fracture with migration or embolization of catheter material [5]. Despite the many complications that may arise, a hemothorax is more likely to ensue following subclavian catheterization as opposed to internal jugular catheterization [6]. Here, we report a case of a large right-sided hemothorax in a patient with breast cancer following ultrasound-guided internal jugular vein cannulation after Port-a-Cath placement.

## Case presentation

A 64-year-old Hispanic female (height 1.524 m; weight 98 kg) with a history of left breast invasive ductal carcinoma grade 2, stage III, presented for an elective ultrasound and fluoroscopic guided Port-a-Cath surgical placement. The procedure was performed in order to facilitate neoadjuvant chemotherapy infusions in the management of her breast cancer. Preoperative evaluation revealed body habitus of morbid obesity (BMI 42 kg/m^2^) and a short neck.

Prior to surgery, the patient was adequately sedated and monitored. The patient was placed in a slight Trendelenburg position. Anatomical landmarks were identified, and a local anesthetic agent was injected into the skin and subcutaneous tissues at the apex of the sternal and clavicular heads of the sternocleidomastoid muscle. Using ultrasound guidance, the right internal jugular vein was identified. A 21-gauge micropuncture needle was used to gain access to the right internal jugular vein, high at the apex of the sternal and clavicular heads of the sternocleidomastoid muscle. By means of the Seldinger technique, a micro guidewire (J tip, 3 mm radius) (Bard Access Systems Inc., Salt Lake City, USA) was then positioned through the needle, and its placement in the right internal jugular vein was confirmed with ultrasound guidance. Under fluoroscopic guidance, the micro guidewire was advanced; however, kinking of the guidewire was noticed at the subclavian and internal jugular vein junction with the wire advanced into the right atrium. Once the wire was placed into the superior vena cava, serial dilators were used to dilate the tract, and a sheath was placed over the wire. This was followed by an unsuccessful attempt to advance the catheter (No. 6 French) (Bard Access Systems Inc., Salt Lake City, USA) through the sheath, as there was difficulty feeding the catheter into the superior vena cava. The sheath and catheter were removed at that time and pressure was held at the site. Another micropuncture was repeated at the apex of the sternal and clavicular heads of the sternocleidomastoid muscle followed by the insertion of a micro guidewire using the Seldindger technique. The micro guidewire entered easily into the right atrium after which a dilator, subsequent sheath, and introducer passed easily as well. The catheter was easily advanced through the sheath the second time without any complications. Under fluoroscopic guidance, the tip of the catheter was positioned at the right cavoatrial junction.

**Figure 1:**
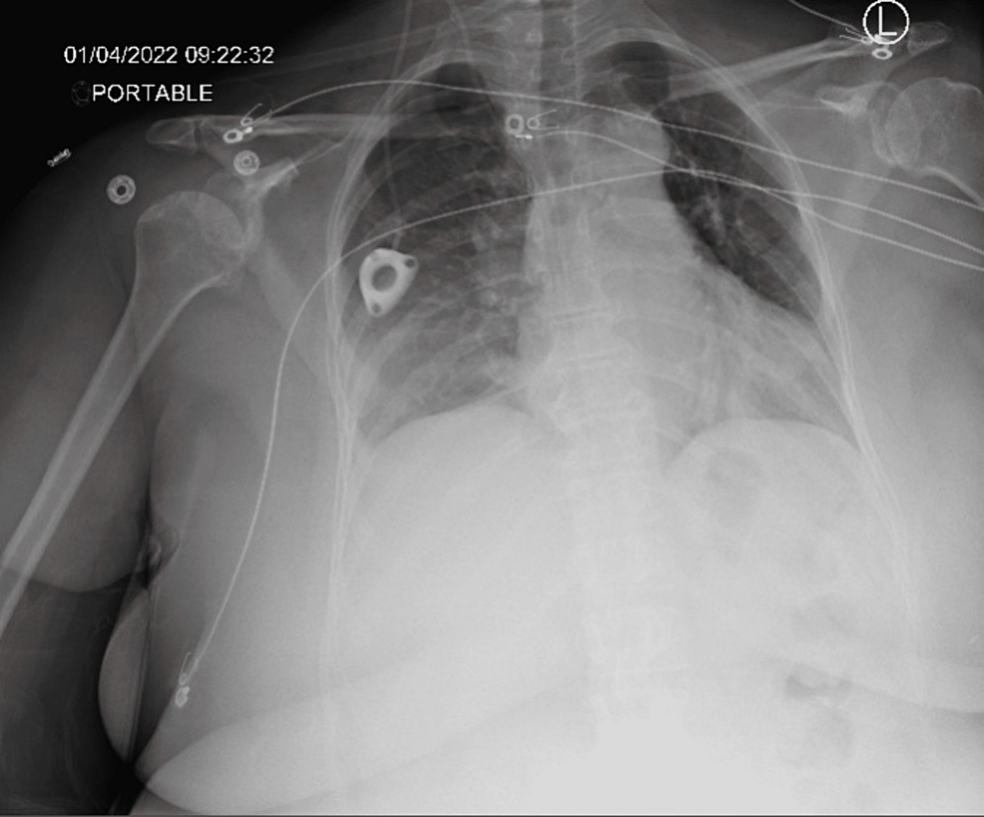
Initial postoperative chest radiograph The image shows a right internal jugular port in place with its catheter tip at the level of the cavoatrial junction. No pneumothorax or evidence of significant pleural effusion was noted.

A small pocket was created beneath the clavicle for port placement. The catheter was attached to the tunneling device and the catheter was brought into the subcutaneous pocket. Under fluoroscopic guidance, the catheter was pulled back so that the tip of the catheter was positioned at the right cavoatrial junction. The port (PowerPort Slim Implantable Port; Bard Access Systems Inc., Salt Lake City, USA) was attached to the catheter and the port was securely sutured on the pectoralis muscular fascia. The catheter was evaluated with injectable heparinized saline. There was a good return of blood with aspiration as well as good flow through the catheter when flushed with a 10 ml saline syringe. The skin was then sutured closed and a postoperative chest X-ray was performed. Chest X-ray showed the successful placement of the Port-a-Cath in the right internal jugular vein with the mediport placed at the level of the atriocaval junction with no evidence of pneumothorax or evidence of pleural effusion (Figure 1).

**Figure 2:**
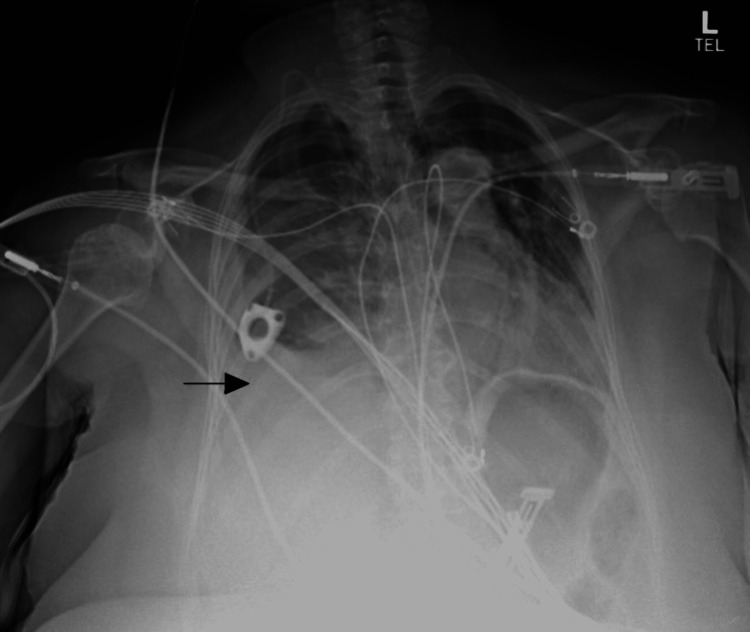
Second postoperative chest radiograph The image shows the right internal jugular mediport again seen with the tip at the level of atriocaval junction. The arrow designates a new small-to-moderate size right pleural effusion/hemothorax.

Prior to discharge, 90 minutes after surgery, the patient was seated in an upright position when she complained of lightheadedness and feeling unwell. The patient then became pale and lethargic after which she was found to be hypotensive with a blood pressure of 72/52 mmHg, tachypneic at 28 breaths per minute, and at a pulse of 70 bpm. Intravenous fluid resuscitation was immediately commenced and a stat chest X-ray was taken. Stat chest X-ray showed a new small to moderate pleural effusion with a concern for a hemothorax (Figure 2). The patient continued to decompensate and required intubation. A central line and an arterial line were placed. She received 2 liters of crystalloid resuscitation and 2 units of packed red blood cells which stabilized her blood pressure.

**Figure 3:**
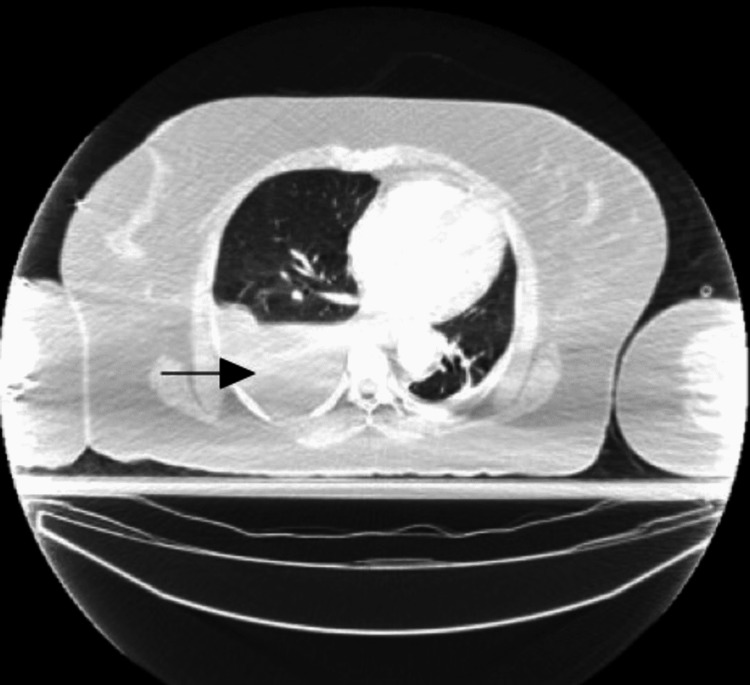
Computed tomographic angiogram of the chest with IV contrast The arrow designates a large right-sided hemothorax.

**Figure 4:**
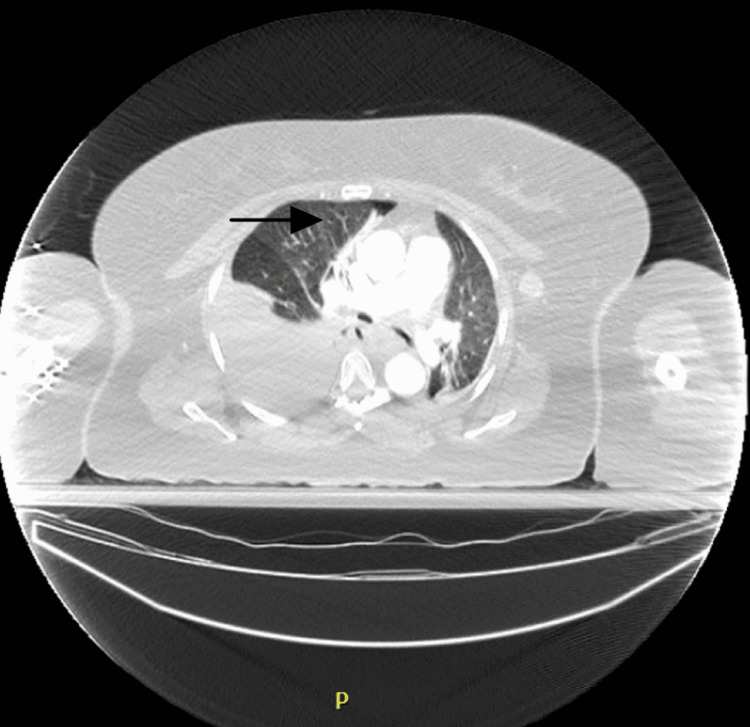
Computed tomographic angiogram of chest with IV contrast The image is again showing the large right-sided hemothorax with a significant mass effect on the right upper lobe and the mediastinal shift to the left.

A computed tomographic angiogram of the chest with IV contrast was obtained which showed a hematoma at the base of the right side of the neck around the recently placed port site. The hematoma measured approximately 3.6 x 3.1 cm in its transverse dimensions. There was no definitive evidence suggestive of arterial injury. There was a large right-sided hemothorax (Figure 3) causing a significant mass effect on the right upper lobe as well as a slight mediastinal shift to the left (Figure 4). The venous phase was not obtained, so identification of active venous bleeding was not definitively identified. There was a growing concern about whether continued venous bleeding would potentially lead to exsanguination if a chest tube was placed to decompress the hemothorax. Due to these concerns, the patient was transferred to a higher acuity center that had cardiothoracic and vascular surgery capabilities.

**Figure 5:**
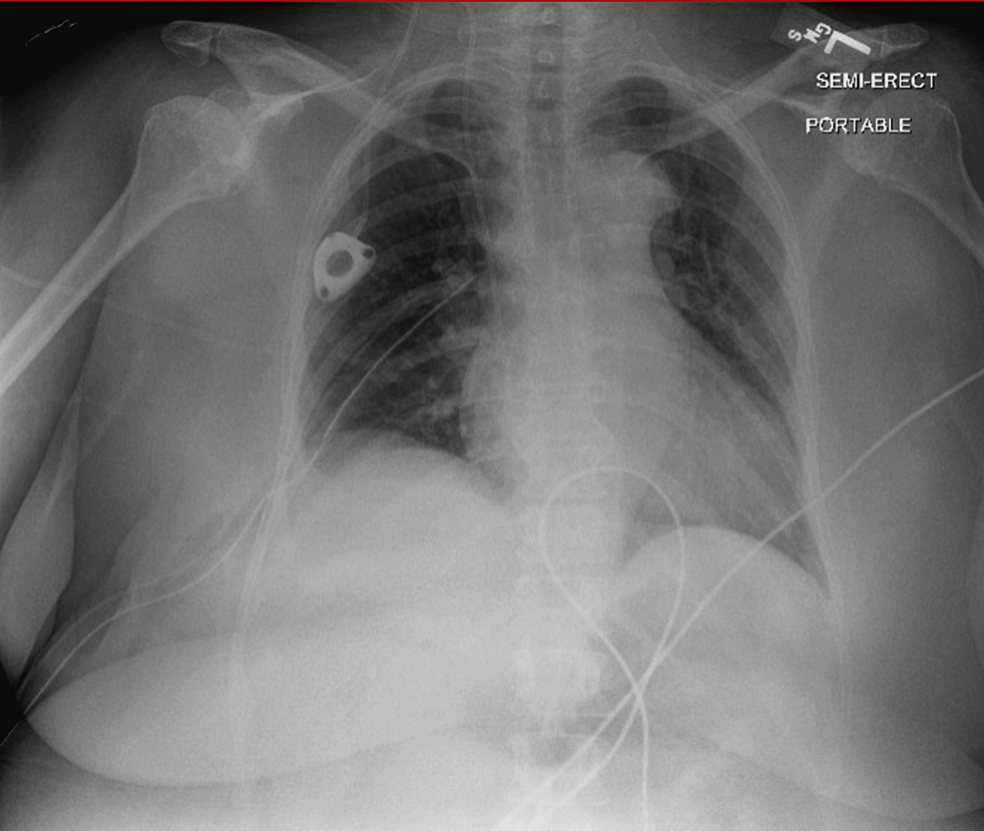
Post VATS chest radiograph VATS: Video-assisted thoracoscopic surgery The image is showing interval placement of the right-sided chest tube with complete resolution of right-sided pleural effusion.

At the tertiary center under management of vascular surgery, the patient underwent a right brachial artery cutdown. An angiogram was obtained which showed a patent innominate, carotid, and subclavian artery with no injuries. The patient then underwent right video-assisted thoracoscopic surgery (VATS) for evacuation of the hemothorax; 800 ml of blood and clot were evacuated. Inspection of the thoracic inlet showed a subpleural hematoma without any active bleeding. A right-sided chest tube was placed after VATS evacuation of the hemothorax (Figure 5). The patient was subsequently transferred to the intensive care unit without any postoperative complications. The remainder of the hospital course was unremarkable and the patient made an uneventful recovery. The patient was discharged in stable condition two days later.

## Discussion

In this case report, we discuss the rare but potentially fatal complication of a hemothorax following internal jugular vein cannulation. Of the various complications that may arise during internal jugular vein cannulation, acute complications include venous rupture as well as arterial punctures, which occur at an incidence of <1%. These acute complications are significant as they can lead to potentially fatal hemothorax formation [2]. Subclavian catheterization is more likely than internal jugular catheterization to be complicated by pneumothorax and hemothorax formation [6]. The rate of pneumothorax and hemothorax after puncture of the subclavian vein ranges from 1.5% to 6% [5]. However, the incidence rate of hemothorax formation following internal jugular cannulation has not been well described in the literature. Ultrasound guidance has been promoted as a method for reducing the risk of complications during central venous catheterization [6]. Specifically, its use in internal jugular venous catheterization has been shown to reduce the number of mechanical complications, catheter-placement failures, and the time required for overall catheter insertion [6]. Despite the promising data on ultrasound guidance and its ability to reduce the incidence of complications associated with internal jugular vein cannulation, the technique alone is insufficient in preventing hemothorax formation, as evident in our case.

Although the cause of the hemothorax was not determined in our patient, we discuss possible theories that may have led to hemothorax formation. First, abnormal vessel anatomy is a risk factor for the potentially fatal complication of hemothorax formation. Recent literature shows that right brachiocephalic vein injury following right internal jugular vein cannulation may be caused by the displacement of the internal jugular vein from a tortuous common carotid artery [3]. Although not confirmed on CT angiogram in our patient, this is one possible cause of hemothorax in our patient. Another possible etiology of hemothorax formation in our patient could have been due to a possible small injury of the internal jugular vein or superior vena cava during guidewire insertion or dilation. This theory is plausible due to the difficulty encountered when advancing the dilator over the guidewire. During insertion of the guidewire, there was some resistance met. This resistance was likely due to the tortuosity of the vessel; thus, the wire was subsequently removed and retrieved in a bent shape. Furthermore, attempts to advance the catheter through the sheath were unsuccessful. We suspect that a possible small injury may have been caused when the guidewire met resistance or when the dilator was used. Thus, the possible tortuous nature of the patient’s vessels could have led to a stiff dilator getting trapped against the vessel wall at the subclavian and internal jugular junction causing vessel injury and leading to the subsequent hemothorax. Contributing risk factors for hemothorax formation in our case could also be attributed to morbid obesity and a short neck length. These known risk factors for hemothorax formation may be attributed to the possible kinking of the dilators, catheters, and sheaths [2].

Upon review of the literature, it is imperative to note the importance of postoperative chest X-rays in order to confirm the adequate placement of internal jugular vein catheters and to screen for possible complications [5]. Postoperative chest X-rays should be obtained in an upright position and in two projections due to the greater degree of diagnostic accuracy when compared to supine radiographs [5]. Park et al. describe different studies that question the use of chest X-rays postoperatively [7]. In one retrospective study, seven years of ultrasound-guided central venous catheter placements were reviewed and it was found that chest radiographs were found to be unnecessary in terms of cost and benefit [7]. In another study, 200 cases were reviewed, in which 198 cases were of internal jugular vein placements, which concluded that routine chest radiographs rarely affected management [7]. It also concluded that the decision to obtain chest radiographs should be guided by clinical factors. This case demonstrates the importance of obtaining routine chest radiographs postoperatively to detect potential complications. The chest X-ray was the initial imaging study obtained which did not show evidence of a hemothorax. Due to clinical concern for bleeding, the chest X-ray was repeated which confirmed a significant right-sided hemothorax. Although this raises some concern and brings into question whether timing should be a factor when obtaining postoperative radiographs, it is reasonable to suggest additional radiographic imaging studies should be obtained in patients that have a complicated postoperative surgical course. Perhaps delaying the initial chest X-ray could have allowed for earlier detection of the hemothorax prior to the patient’s clinical decompensation. More data and research is needed to ascertain whether the timing of radiographic images after central venous catheter insertions could optimize the detection of delayed hemothorax or pneumothorax formation postoperatively.

## Conclusions

In conclusion, although hemothorax formation is a rare complication following internal jugular vein cannulation, it warrants consideration as it has potentially fatal outcomes. Abnormal vessels, body habitus and neck length are a few factors that should be evaluated when assessing possible risks and procedural difficulties. Although the use of ultrasound guidance is now standard of practice and has been shown to reduce the rates of complication associated with internal jugular vein catheter placement, it does not eliminate the possible complication of hemothorax formation. Postoperative chest radiographs are a standard practice in patients that undergo internal jugular vein catheter placement and through this article we advocate for the continued use of this routine imaging modality. However, we also advocate for factors such as the timing of the imaging studies to be further considered and investigated based on clinical criteria and risk factors.
